# Association between minimum nocturnal heart rate and cerebral small vessel disease in patients with obstructive sleep apnea

**DOI:** 10.3389/fnagi.2026.1848124

**Published:** 2026-09-15

**Authors:** Wenqiu Wei, Yining Wen, Fangmei Chen, Ankang Liang, Qingqing Zhong, Xiaoyi Shi, Manchan Wei, Lixia Qin, Yian Lu, Mingming Zhao

**Affiliations:** 1 Department of Sleep Medicine, The People's Hospital of Guangxi Zhuang Autonomous Region, Joint Training Unit of Guangxi University of Chinese Medicine, Nanning, Guangxi, China; 2 Guangxi Research Center for Sleep Medicine, The People's Hospital of Guangxi Zhuang Autonomous Region, Guangxi Medical Academy, Nanning, Guangxi, China

**Keywords:** cerebral small vessel disease, heart rate, obstructive sleep apnea, sleep medicine, sleep structure

## Abstract

**Introduction:**

Obstructive sleep apnea (OSA) has been implicated in cerebral small vessel disease (CSVD) through mechanisms involving intermittent hypoxia, autonomic imbalance, and metabolic dysregulation, but the pathways remain insufficiently elucidated. This retrospective observational study sought to identify clinical and physiological factors associated with CSVD in adults with OSA.

**Methods:**

We reviewed adult patients who underwent overnight polysomnography and were diagnosed with OSA at the Sleep Medicine Department of the People's Hospital of Guangxi Zhuang Autonomous Region from January 2021 to December 2023. Data collected included demographics, polysomnography, biochemical markers, and brain MRI. The primary outcome was the presence of CSVD. Multivariate logistic regression determined independent factors, and model discrimination was assessed with the area under the receiver operating characteristic curve (AUC).

**Results:**

Among 630 patients, 265 (42%) had CSVD. Compared with those without CSVD, patients with CSVD had shorter NREM sleep latency, more nocturnal awakenings, and a distinct lipid profile with lower LDL-C and ApoB, but higher HDL-C and ApoA1/B ratios. Multivariate analysis identified older age (OR 1.095, 95% CI 1.071–1.118, *p* < 0.001) and higher nocturnal minimum heart rate (OR 1.023, 95% CI 1.006–1.040, *p* = 0.008) as independently associated with CSVD. The model showed good discrimination (AUC 0.752).

**Discussion:**

Elevated nocturnal minimum heart rate, possibly reflecting altered autonomic regulation, was independently associated with CSVD in OSA. Findings suggest autonomic dysfunction and sleep fragmentation may contribute to CSVD pathogenesis in this population.

## 1 Introduction

Obstructive sleep apnea (OSA) is a prevalent sleep-related breathing disorder characterized by recurrent upper airway collapse, leading to intermittent hypoxia, sleep fragmentation, and autonomic dysregulation (Lv et al., 2023; Yeghiazarians et al., 2021). With the growing global burden of obesity, the prevalence of OSA has markedly increased. OSA is recognized as a significant risk factor for both cardiovascular and cerebrovascular diseases, and emerging evidence has suggested its potential association with cerebral small vessel disease (CSVD; Gottlieb and Punjabi, 2020; Ward et al., 2025). CSVD, characterized by white matter hyperintensities (WMHs), lacunes, and cerebral microbleeds, represents a subclinical marker of brain injury associated with cognitive decline and increased stroke risk (Hotz et al., 2021; Mahammedi et al., 2022).

Clinical and neuroimaging studies have indicated that patients with OSA frequently exhibit microvascular alterations in the brain, which may be driven by intermittent hypoxia, blood pressure variability, and heightened sympathetic activity (Mao et al., 2024). However, the precise mechanisms linking OSA-related physiological disturbances to CSVD remain insufficiently understood. Identifying specific factors associated with CSVD among patients with OSA could help elucidate the underlying pathophysiological pathways and improve cerebrovascular risk stratification.

Several cross-sectional and longitudinal studies have reported a greater CSVD burden or increased WMH volume in patients with moderate-to-severe OSA compared with healthy controls (Lee et al., 2021; Narkiewicz et al., 1998; Qin et al., 2021). In these individuals, autonomic imbalance and cardiovascular dysregulation may further exacerbate cerebrovascular injury (Maniaci et al., 2024). Nocturnal heart rate—particularly the minimum heart rate during sleep—serves as a reliable indicator of parasympathetic recovery and overall autonomic stability. Previous population-based and hospital-based studies have demonstrated that elevated nocturnal or minimum heart rate is associated with a greater burden of WMHs and an increased risk of stroke or CSVD (Amiya et al., 2014; Mancia et al., 2021; Yugar et al., 2023). Nevertheless, few investigations have examined this relationship specifically within OSA populations, in whom autonomic disturbances are especially pronounced. Understanding whether the minimum nocturnal heart rate independently correlates with CSVD may help identify high-risk OSA patients and refine early cerebrovascular risk assessment (Zhang et al., 2021).

Therefore, this study aimed to identify factors associated with CSVD in patients with OSA, with particular attention to autonomic dysfunction, sleep architecture, and biochemical parameters. By elucidating the potential determinants of CSVD in OSA, this research seeks to provide insights that may enhance prevention, diagnosis, and individualized management strategies for affected patients.

## 2 Methods

### 2.1 Study design and setting

This retrospective observational study was conducted in the Sleep Medicine Department of the People's Hospital of Guangxi Zhuang Autonomous Region between January 2021 and December 2023. Relevant data were retrieved from the hospital's electronic medical record system and the polysomnography (PSG) database. The study protocol was reviewed and approved by the Institutional Review Board of the People's Hospital of Guangxi Zhuang Autonomous Region (Ethics Approval No. KY-GJZDYF-2023-03).

### 2.2 Study participants

Eligible participants were adults aged 18–65 years who were diagnosed with OSA based on the diagnostic criteria outlined in the American Academy of Sleep Medicine (AASM) Manual, Version 2.6 (Berry et al., 2020), defined as an apnea–hypopnea index (AHI) ≥5 events per hour on PSG accompanied by relevant clinical symptoms (e.g., excessive daytime sleepiness, fatigue, or witnessed apneas). Patients were excluded if they had comorbid insomnia; a history of stroke, atrial fibrillation, heart failure, or chronic kidney disease; or major systemic diseases such as malignancy, trauma, autoimmune, neurological, or other severe disorders; or if they declined to complete MRI examinations and biochemical testing (Figure 1). For all enrolled participants, MRI and biochemical testing were conducted as part of the standard inpatient workup. All MRI examinations were performed within 1 week of hospital admission, in the same week as polysomnography.

**Figure 1:**
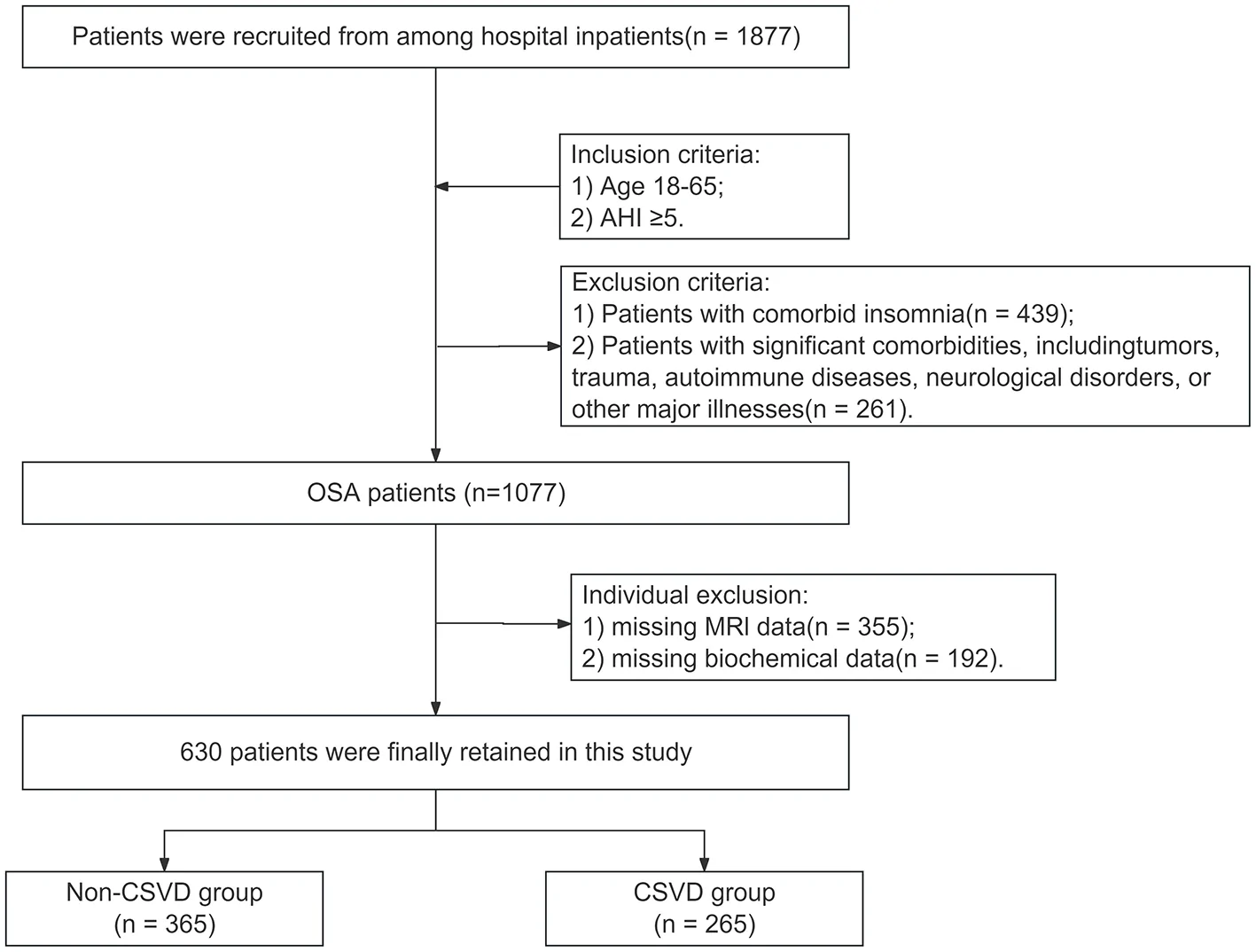
Flow chart for the OSA patients who were included in the analysis.

The primary outcome was the presence of CSVD, defined according to the Standards for Reporting Vascular Changes on Neuroimaging (STRIVE) criteria. CSVD was diagnosed when one or more of the following MRI findings were present: white matter hyperintensities, lacunes, cerebral microbleeds, enlarged perivascular spaces, or brain atrophy. Based on these findings, participants were categorized into two groups: the CSVD group and the non-CSVD group.

### 2.3 Data collection

Clinical, biochemical, and polysomnographic data were extracted from electronic records. Demographic and clinical variables included age, sex, body mass index (BMI), marital status, smoking and drinking history, hypertension, diabetes, arteriosclerosis, and medication use (e.g., statins, beta-blockers). Biochemical parameters included total cholesterol (TC), triglycerides (TG), low-density lipoprotein cholesterol (LDL-C), high-density lipoprotein cholesterol (HDL-C), apolipoprotein A1 (ApoA1), apolipoprotein B (ApoB), urea, uric acid, creatinine, and liver function indicators [alanine aminotransferase (ALT), aspartate aminotransferase (AST), albumin (ALB), total protein (TP), cholinesterase (ChE), and total bile acids (TBA)]. Polysomnographic parameters included total sleep time, sleep onset latency, non-rapid eye movement (NREM) and rapid eye movement (REM) sleep latency, number of awakenings, wake time after sleep onset, periodic limb movement (PLM) index, apnea–hypopnea index (AHI), and the minimum nocturnal heart rate, defined as the lowest heart rate recorded during the PSG examination. All polysomnographic recordings were performed in the Sleep Medicine Department using a Compumedics E-Series EEG/PSG system (Compumedics, Australia) and scored according to the AASM Manual, Version 2.6, by two independent technologists. Discrepancies were resolved by consensus.

### 2.4 Neuroimaging assessment

Brain MRI was performed on a 3.0 T scanner. Sequences included T1-weighted, T2-weighted, FLAIR. All MRI examinations were completed within 1 week of admission, in the same week as polysomnography. Images were independently assessed by two radiologists blinded to polysomnographic and clinical data, and discrepancies were resolved by consensus. White matter hyperintensity severity was graded using the Fazekas scale. Patients with prior stroke, acute infarcts, atrial fibrillation, heart failure, chronic kidney disease, or relevant neurological disease were excluded.

### 2.5 Sample size calculation

The required sample size was estimated using the rule of at least 10 events per variable (EPV) to ensure reliable logistic regression modeling. Assuming seven independent variables and a CSVD prevalence of approximately 46.8% among OSA patients (Nishibayashi et al., 2008), the minimum required sample size was 150 participants. This criterion was met and exceeded, as the final cohort included 630 patients, comprising 265 with CSVD (cases) and 365 without CSVD (controls).

### 2.6 Statistical analysis

All statistical analyses were performed using R software (version 4.0.3; R Foundation for Statistical Computing, Vienna, Austria) and Python (version 3.13; Python Software Foundation, Wilmington, DE, USA). Continuous variables were presented as mean ± standard deviation (SD) for normally distributed data or as median (interquartile range) for non-normally distributed data. Between-group comparisons were conducted using the t-test or Mann–Whitney U test, as appropriate. Categorical variables were expressed as counts and percentages, and compared using the chi-square test. Candidate variables for multivariate logistic regression were selected based on: (1) established risk factors for CSVD or cerebrovascular disease reported in previous studies (e.g., age, sex, hypertension, diabetes, and smoking; Liang et al., 2023; Liu et al., 2018; Mkhitaryan and Fateeva, 2024; Zhang et al., 2025); and (2) variables showing significant differences (*p* < 0.05) between the CSVD and non-CSVD groups in univariate analyses. Before model construction, multicollinearity among independent variables was evaluated using variance inflation factors (VIFs). Variables with VIF >10 were considered to exhibit substantial collinearity and were either excluded or represented by the most clinically relevant variable from correlated clusters. Model discrimination was assessed by the area under the receiver operating characteristic curve (AUC), and calibration was evaluated using the Hosmer–Lemeshow goodness-of-fit test and calibration plots. A two-tailed *p*-value < 0.05 was considered statistically significant.

## 3 Results

### 3.1 Participant characteristics

A total of 630 patients with OSA were included, with a mean age of 44.43 ± 10.75 years. Among them, 265 (42%) were diagnosed with CSVD. Baseline demographic and clinical characteristics stratified by CSVD status are summarized in Table 1. Compared with patients without CSVD, those with CSVD were older and had a higher prevalence of marital status, hypertension, and arteriosclerosis or arterial plaque (all *p* < 0.05). No significant between-group differences were observed in the distribution of specific imaging markers. Among the 265 patients with CSVD, white matter hyperintensities were the predominant MRI finding (94.3%), followed by lacunes (5.7%) and brain atrophy (5.3%). Enlarged perivascular spaces (1.9%), cerebral microbleeds (1.1%), and recent small subcortical infarcts (0.8%) were less frequently observed (Table S1). WMH severity graded by the Fazekas scale was grade 0 in 12 patients (4.5%), grade 1 in 239 (90.2%), grade 2 in 13 (4.9%), and grade 3 in 1 (0.4%).

**Table 1:** Baseline characteristics of participants.

Characteristics	Overall (*n* = 630)	Non-CSVD group (*n* = 365)	CSVD group (*n* = 265)	*P*
Age (mean ± SD)	44.43 ± 10.75	40.74 ± 9.95	49.52 ± 9.71	< 0.001
Sex (*n*, %)				0.115
Female	94 (14.92)	47 (12.88)	47 (17.74)	
Male	536 (85.08)	318 (87.12)	218 (82.26)	
BMI (mean ± SD)	27.61 ± 4.19	27.69 ± 4.40	27.50 ± 3.88	0.579
Nation (*n*, %)				0.637
Minority	228 (36.48)	135 (37.40)	93 (35.23)	
Han	397 (63.52)	226 (62.60)	171 (64.77)	
CHD (*n*, %)	9 (1.44)	3 (0.83)	6 (2.27)	0.248
Hyperlipemia (*n*, %)	56 (8.96)	33 (9.14)	23 (8.71)	0.965
Smoking (*n*, %)	114 (18.24)	68 (18.84)	46 (17.42)	0.729
Drinking (*n*, %)	152 (24.32)	91 (25.21)	61 (23.11)	0.610
Married status (*n*, %)	518 (82.88)	282 (78.12)	236 (89.39)	< 0.001
Hypertension (*n*, %)	188 (30.08)	89 (24.65)	99 (37.50)	0.001
Diabetes (*n*, %)	46 (7.36)	20 (5.54)	26 (9.85)	0.060
Arteriosclerosis/Arterial plaque (*n*, %)	127 (24.85)	48 (16.16)	79 (36.92)	< 0.001

CSVD, cerebral small vessel disease; BMI, body mass index; CHD, coronary heart disease.

### 3.2 Sleep architecture and polysomnographic findings

Significant group differences were observed in polysomnographic parameters (Figures 2B, C, Table 2). Patients with CSVD demonstrated a higher nocturnal minimum heart rate (*p* = 0.009), which was positively correlated with the apnea–hypopnea index (Figure 4). They also exhibited more frequent nocturnal awakenings (*p* = 0.023) and a higher periodic limb movement (PLM) index during REM sleep (*p* = 0.022). In addition, the CSVD group showed shorter sleep onset latency across multiple NREM stages, including stage N1 (*p* = 0.001), stage N2 (*p* = 0.012), and stage N3 (*p* = 0.006), indicating more pronounced sleep fragmentation.

**Figure 2:**
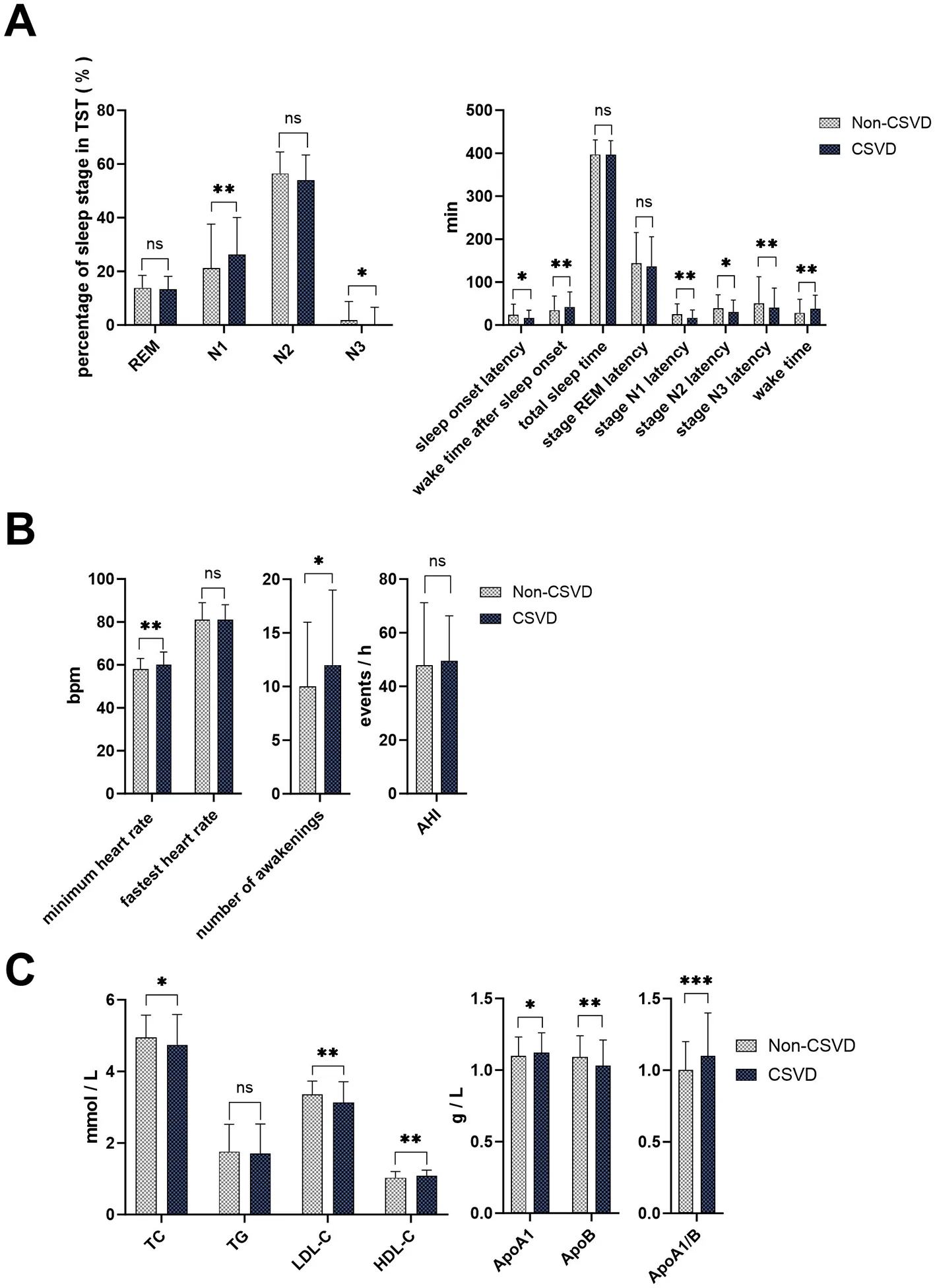
Comparison of lipid metabolism and PSG parameters between non-CSVD and CSVD groups. **(A)** Analysis of lipid metabolism levels: including the levels of total cholesterol (TC), triglycerides (TG), low-density lipoprotein cholesterol (LDL-C), high-density lipoprotein cholesterol (HDL-C), apolipoprotein A1 (ApoA1), apolipoprotein B (ApoB), and the ApoA1/ApoB ratio. **(B)** Comparative analysis of sleep-related events: including the number of awakenings, periodic limb movement (PLM) index in REM sleep, and apnea-hypopnea index (AHI). **(C)** Analysis of Sleep Architecture: including the percentage of time spent in each sleep stage (REM, N1, N2, and N3) and the duration of sleep onset latency, total sleep time, REM sleep time, and wake time after sleep onset (*P < 0.05, **P < 0.01, ***P < 0.001).

**Table 2:** Comparison of polysomnography parameters between Non-CSVD and CSVD groups.

Variables	Non-CSVD group	CSVD group	*P*
Sleep onset latency (min)	23.50 [9.50, 48.50]	16.00 [8.50, 34.50]	0.005
Wake time after sleep onset (min)	34.50 [16.00, 67.50]	42.00 [21.50, 77.00]	0.009
Sleep efficiency (%)	84.80 [76.90, 91.80]	85.10 [76.60, 91.20]	0.736
Number of awakenings	10.00 [5.00, 16.00]	12.00 [6.00, 19.00]	0.023
Total sleep time (min)	397.00[359.00, 431.00]	396.00[355.50, 429.00]	0.735
Stage REM latency (min)	144.00[97.00, 215.50]	136.50 [91.50, 205.50]	0.284
REM (%)	13.80 [9.20, 18.50]	13.30 [9.20, 18.10]	0.522
Stage N1 latency (min)	25.00 [11.00, 49.50]	17.00 [9.50, 35.00]	0.001
N1 (%)	21.30 [13.60, 37.60]	26.30 [17.50, 40.10]	0.007
Stage N2 latency (min)	39.50 [21.00, 70.50]	30.50 [17.00, 58.00]	0.012
N2 (%)	56.50 [42.80, 64.50]	54.00 [43.10, 63.40]	0.229
Stage N3 latency (min)	51.00 [24.00, 112.50]	40.00 [14.00, 86.00]	0.006
N3 (%)	1.70 [0.00, 8.80]	0.00 [0.00, 6.60]	0.016
Wake time (min)	28.00 [12.50, 60.00]	37.50 [18.50, 69.50]	0.001
Minimum heart rate (bpm)	58.00 [52.00, 63.00]	60.00 [53.00, 66.00]	0.009
Fastest heart rate (bpm)	81.00 [74.00, 89.00]	81.00 [73.00, 88.00]	0.713
Average SpO_2_ in awake time (%)	96.00 [95.00, 96.00]	96.00 [95.00, 96.00]	0.142
The lowest SpO_2_ (%)	73.00 [60.00, 82.00]	73.00 [61.00, 81.00]	0.787
Average SpO_2_ desaturation (%)	7.00 [5.00, 12.00]	7.00 [5.00, 10.00]	0.572
Number of arousals caused by respiratory events during REM sleep	7.00 [2.00, 17.00]	6.00 [2.00, 15.00]	0.397
Number of arousals caused by limb movement during REM sleep	0.00 [0.00, 0.00]	0.00 [0.00, 0.00]	0.020
PLM index in stage REM	0.00 [0.00, 18.30]	0.00 [0.00, 8.60]	0.022
AHI (events/h)	47.90 [27.40, 71.20]	49.40 [30.50, 66.30]	0.583

TST, total sleep time; REM, rapid eye movement, REM, N1, N2, and N3%: the percentage of N1 period, N2 period, N3 period and REM period in total sleep time, PLM, periodic leg motion; AHI, apnea hypopnea index.

### 3.3 Biochemical and medication profiles

Distinct biochemical patterns were identified between groups (Figure 2A, Table 3). Compared with the non-CSVD group, patients with CSVD exhibited lower levels of TC, LDL-C, and ApoB, but higher levels of HDL-C, ApoA1, ApoA1/ApoB ratio, and urea (all *p* < 0.05).

**Table 3:** Comparison of biochemical indexes between Non-CSVD and CSVD groups.

Variables	Non-CSVD group	CSVD group	*P*
TC (mmol/L)	4.95 [4.34, 5.57]	4.74 [4.04, 5.59]	0.027
TG (mmol/L)	1.76 [1.27, 2.52]	1.70 [1.20, 2.53]	0.345
LDL-C (mmol/L)	3.35 [2.88, 3.73]	3.13 [2.55, 3.71]	0.007
HDL-C (mmol/L)	1.03 [0.93, 1.20]	1.08 [0.95, 1.24]	0.037
ApoA1 (g/L)	1.10 [0.97, 1.23]	1.12 [1.01, 1.26]	0.017
ApoB (g/L)	1.09 [0.91, 1.24]	1.03 [0.81, 1.21]	0.004
ApoA1/B	1.00 (0.90, 1.20)	1.10 (0.90, 1.40)	< 0.001
Urea (mmol/L)	4.90 [4.24, 5.62]	5.20 [4.50, 6.00]	0.001
CREA (umol/L)	80.16 [71.03, 90.00]	82.82 [72.00, 91.47]	0.317
UA (umol/L)	439.00 [376.00, 514.02]	420.00 [359.80, 491.20]	0.048
ALT (U/L)	26.60 [18.67, 47.00]	22.75 [16.22, 34.20]	< 0.001
AST (U/L)	23.00 [19.32, 29.20]	21.50 [19.00, 26.00]	0.004
TP (g/L)	68.30 [65.40, 71.45]	67.60 [64.40, 71.20]	0.043
ALB (g/L)	41.68 [39.88, 43.75]	40.94 [38.76, 43.06]	0.001
ChE (U/L)	9228.00 [8269.90, 10366.00]	8637.25 [7866.35, 9940.77]	0.001
TBA (umol/L)	3.40 [2.20, 6.30]	3.10 [1.90, 5.18]	0.031

TC, total cholesterol; TG, triglyceride; LDL-C, low-density lipoprotein cholesterol; HDL-C, high-density lipoproteincholesterol; ApoA1, apolipoprotein A1; ApoB, apolipoprotein B; CREA, creatinine; UA, uric acid; ALT, alanine aminotransferase; AST, aspartate aminotransferase; TP, total protein; ALB, albumin; ChE, cholinesterase; TBA, total bile acids.

Additionally, the CSVD group had lower uric acid levels (*p* = 0.048) and reduced liver function indicators, including ALT (*p* < 0.001), AST (*p* = 0.004), TP (*p* = 0.043), ALB (*p* = 0.001), ChE (*p* = 0.001), and TBA (*p* = 0.031).

Medication analysis showed that statin use was more common in the CSVD group than in the non-CSVD group (*p* = 0.003; Table 4).

**Table 4:** Comparison of drug use between Non-CSVD and CSVD groups.

Medication	Non-CSVD group	CSVD group	*P*
Statin use(%)	37 (10.14)	50 (18.87)	0.003
Beta-blocker use(%)	16 (4.38)	18 (6.79)	0.253

### 3.4 Association of nocturnal minimum heart rate with CSVD risk

Multicollinearity was assessed using VIFs, and all variables in multivariate logistic regression model demonstrated acceptable independence (VIF < 2.1; Table S3). Multivariate logistic regression identified older age (OR = 1.095, 95% CI: 1.071–1.118, *p* < 0.001) and elevated nocturnal minimum heart rate (OR = 1.023, 95% CI: 1.006–1.040, *p* = 0.008) as independent factors associated with the presence of CSVD (Table 5).

**Table 5:** Multivariate logistic regression analysis of factors associated with CSVD in OSA patients.

Variables	OR	95% *CI*	*P*
Minimum heart rate	1.023	1.006–1.040	0.008
Age	1.095	1.071–1.118	< 0.001
Sex	1.324	0.784–2.233	0.294
Marital status	0.935	0.541–1.615	0.809
Smoke	1.071	0.673–1.704	0.774
Statin use	1.198	0.706–2.034	0.504
Beta-blocker use	0.922	0.407–2.088	0.846
Diabetes	1.038	0.524–2.058	0.915
Hypertension	1.119	0.740–1.692	0.593

In a sensitivity analysis excluding statin users (*n* = 542), higher minimum nocturnal heart rate remained independently associated with CSVD (OR 1.021, 95% CI 1.003–1.039, *p* = 0.019), consistent with the primary model (Table S4). When overall AHI was added as a continuous covariate, the association was essentially unchanged (OR 1.022, 95% CI 1.004–1.039, *P* = 0.014), whereas AHI itself was not significant (OR 1.003, 95% CI 0.995–1.010, *P* = 0.482), indicating that the association was not explained by OSA severity. Descriptive stratification by OSA severity is provided in Table S5 and the AHI-adjusted model in Table S6.

### 3.5 Predictive performance of nocturnal minimum heart rate for CSVD

The predictive model demonstrated good performance, with an AUC of 0.752 (Figure 3) and satisfactory calibration as indicated by the Hosmer–Lemeshow goodness-of-fit test (χ^2^ = 4.383, *p* = 0.821; Figure 5).

**Figure 3:**
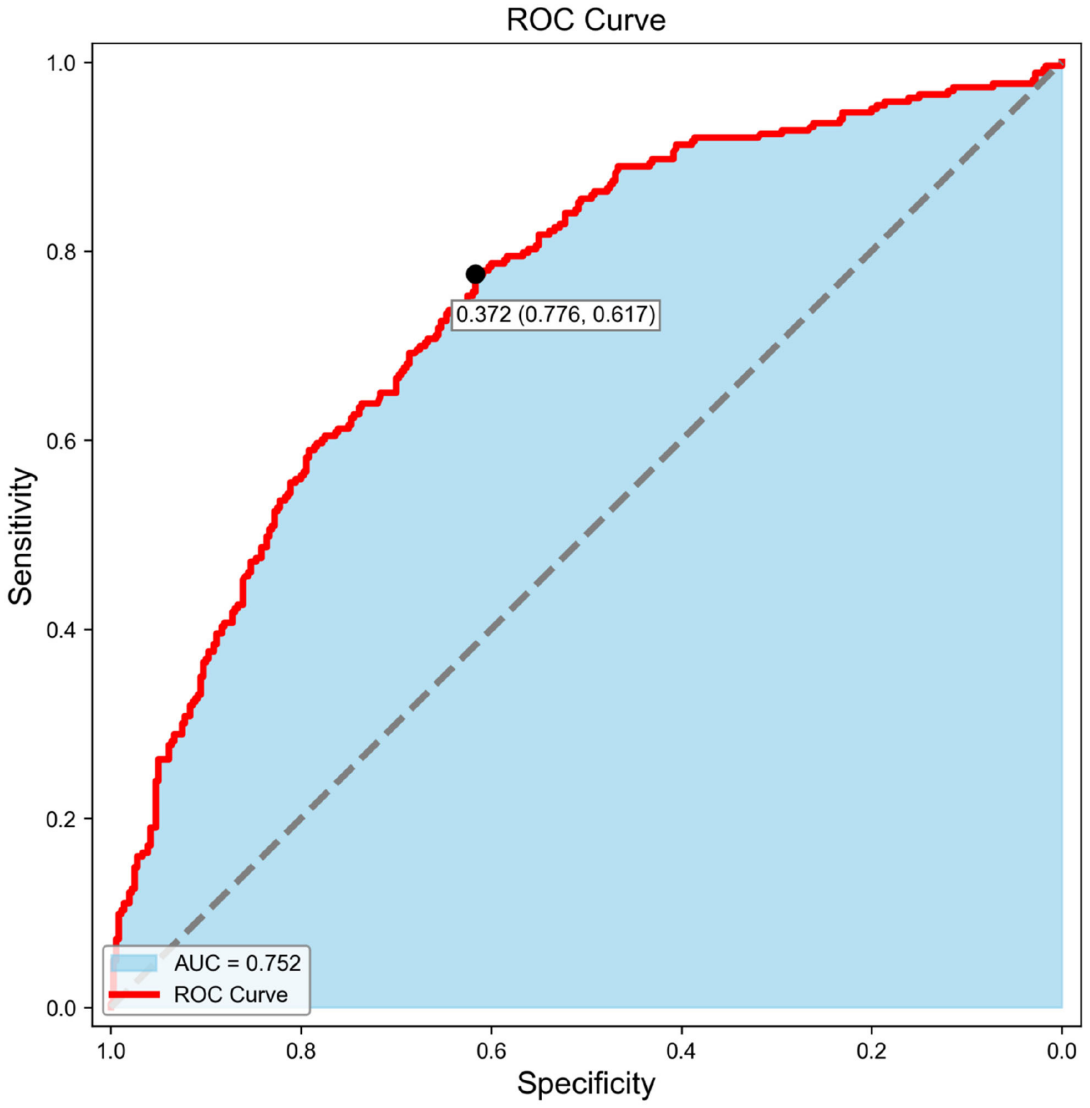
ROC curve of multivariate logistic regression model.

**Figure 4:**
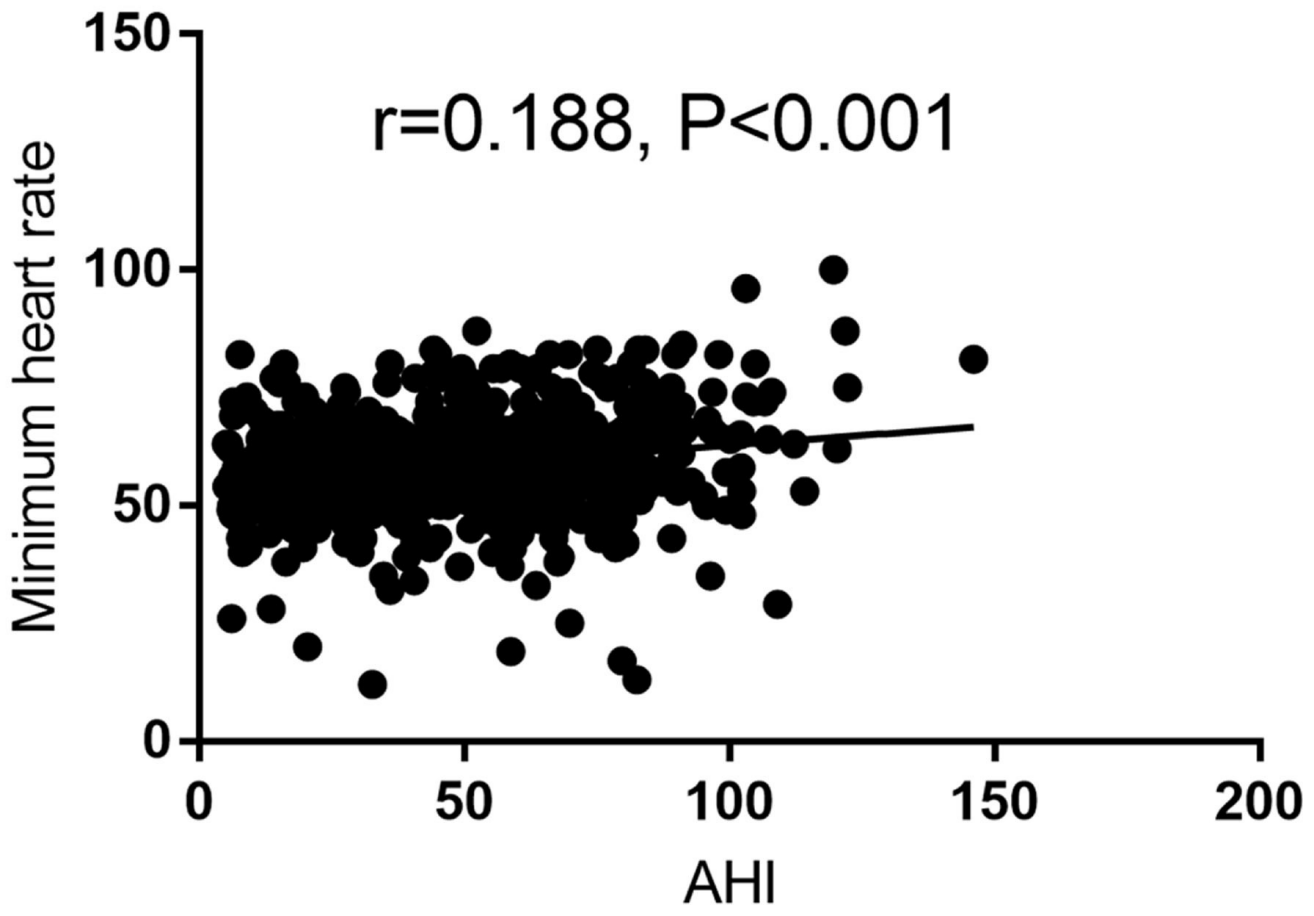
Correlation analysis between AHI and minimum heart rate.

**Figure 5:**
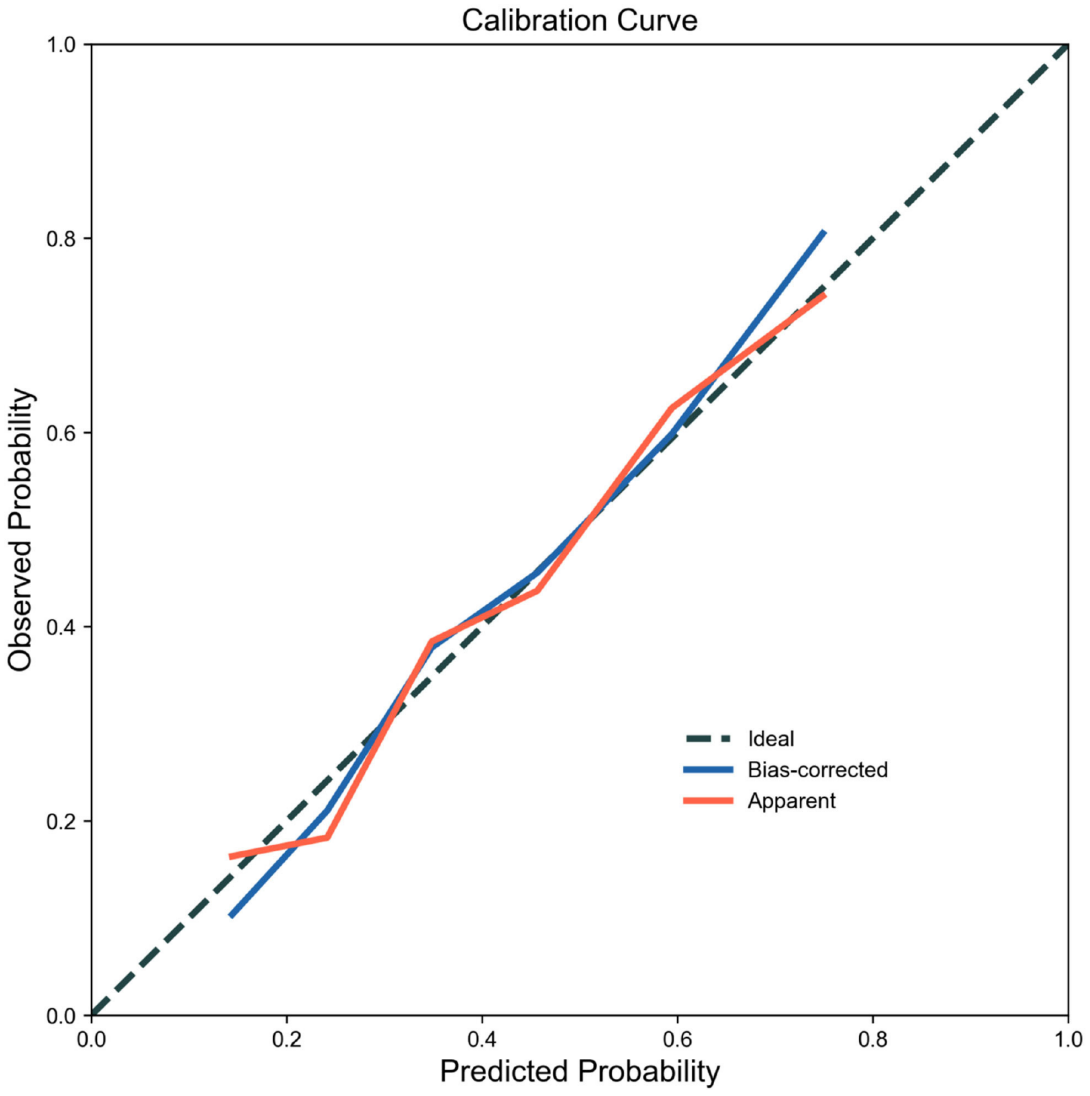
Calibration curve of the multivariate logistic regression model.

## 4 Discussion

This study found that a higher nocturnal minimum heart rate was independently associated with CSVD in patients with OSA. This suggests that persistent nocturnal autonomic activation may increase cerebrovascular vulnerability. In addition, patients with CSVD showed more frequent awakenings, shorter NREM sleep latency, and distinct lipid profile changes, indicating multifactorial involvement of autonomic, sleep, and metabolic pathways. Clinically, monitoring nocturnal heart rate may help identify OSA patients at higher risk for subclinical cerebrovascular injury.

The association between elevated nocturnal minimum heart rate and CSVD underscores the pivotal role of autonomic dysfunction in OSA-related cerebrovascular pathology. Recurrent apneic events in OSA induce intermittent hypoxia and arousals, activating chemoreflexes and baroreflexes that elevate heart rate and blood pressure (DiCaro et al., 2024). This sustained autonomic imbalance may contribute to endothelial dysfunction and microvascular damage, key precursors of CSVD (Amiya et al., 2014; Durgan and Bryan, 2012). Our multivariate analysis confirms minimum heart rate as an independent risk factor, after adjusting for age, sex, hypertension, diabetes, smoking, and medication use (statins, beta-blockers), extending prior evidence linking heart rate variability (HRV) to OSA severity and cardiovascular outcomes (Qin et al., 2021), suggesting that nocturnal minimum heart rate could serve as a practical, non-invasive marker for identifying OSA patients at risk for cerebrovascular complications. Notably, the association between minimum nocturnal heart rate and CSVD remained essentially unchanged after adjustment for overall AHI, whereas AHI itself was not independently associated with CSVD, suggesting that nocturnal heart rate, as an index of autonomic regulation, may capture cerebrovascular risk information beyond that conveyed by conventional measures of OSA severity. These findings align with Yu et al.'s (2021) study of 778 hospitalized CSVD patients, which demonstrated that each 1 bpm increase in nighttime heart rate was independently associated with a 4.1% higher odds of being in the highest white matter hyperintensity volume quartile. Their results support the notion that elevated nocturnal heart rate reflects sustained sympathetic activation, which may promote microvascular damage and white-matter deterioration. Similarly, the prospective Japan Ambulatory Monitoring study of 6,359 participants showed that a 10-bpm increase in nighttime heart rate was associated with 36%−47% higher risk of total cardiovascular events over 4.5 years of follow-up, independent of blood pressure measures (Kario et al., 2023). Further supporting the autonomic dysfunction hypothesis, Del Brutto et al. (2022) found that decreased nighttime heart rate variability was significantly associated with white matter hyperintensity progression in community-dwelling older adults in their prospective longitudinal study. The clinical relevance of these autonomic measures is underscored by recent meta-analytic evidence showing moderate-to-severe OSA confers nearly four-fold higher odds of white matter hyperintensities compared to no OSA (Lee et al., 2023).

Sleep architecture disruptions, including increased awakenings, reduced sleep onset latency across N1–N3 stages, and elevated PLM indices during REM sleep, were significantly more prevalent in OSA patients with CSVD (Cristini et al., 2025). These disruptions in sleep architecture are linked to sympathetic activation and blood pressure variability, which may exacerbate vascular damage (Chouchou et al., 2013; Lai et al., 2016). Several epidemiological studies have identified OSA as a risk factor for systemic hypertension (Ahmed et al., 2023; Li et al., 2023; Yao et al., 2023), but a direct etiologic link between the two disorders has not been established definitively. The demonstration that OSA can lead to the development of sustained hypertension has considerable importance, given the high prevalence of both disorders in the population (Kwon et al., 2024). These findings underscore the need for comprehensive polysomnographic evaluations to detect sleep structure changes that heighten CSVD risk, aligning with prior research on sleep disruption and vascular health.

Unexpectedly, the CSVD group exhibited lower TC, LDL-C, and ApoB, alongside elevated HDL-C, ApoA1, and ApoA1/ApoB ratio, diverging from conventional cardiovascular risk profiles (Liang et al., 2023). Our paradoxical lipid findings contrast with the conventional OSA literature, where patients typically exhibit atherogenic dyslipidemia characterized by elevated triglycerides and reduced HDL-C levels (Fang et al., 2023). However, emerging evidence suggests that lipid-cerebrovascular relationships are more complex than traditional cardiovascular risk models predict (Potenza et al., 2023; Sergi et al., 2023). In a large UK Biobank metabolomics study of 118,021 participants (including 10,024 with brain MRI data), lower apolipoprotein concentrations were paradoxically associated with greater white-matter microstructural damage, while larger and very-large HDL particles were linked to increased white-matter injury and higher stroke risk, contradicting the conventional HDL-protective paradigm (Harshfield and Markus, 2023). We speculate that these lipid alterations represent a compensatory response to OSA-induced oxidative stress and inflammation, potentially modulated by autonomic dysfunction and sleep fragmentation; however, this interpretation remains speculative and requires further investigation (Marinelli et al., 2025; Meszaros and Bikov, 2022; Pownall et al., 2021). However, the mechanisms underlying this paradoxical lipid profile remain elusive, necessitating further investigation to determine whether these changes are adaptive or indicative of broader metabolic dysregulation. These contrasting findings highlight the need for lipoprotein subfraction analysis and functional assessments beyond standard lipid panels to understand the complex interplay between OSA, lipid metabolism, and cerebrovascular pathology.

Age emerged as a significant risk factor for CSVD, consistent with its established role in cerebrovascular disease (Lee et al., 2023). Notably, the mean age of our CSVD cohort was younger than in previous studies, suggesting that OSA may accelerate cerebrovascular aging through synergistic effects of autonomic dysfunction, sleep fragmentation, and metabolic dysregulation. Confounders such as hypertension and arteriosclerosis, more prevalent in the CSVD group, were adjusted for in our analysis but likely contribute to the observed associations. Hypertension, highly prevalent in OSA, exacerbates endothelial dysfunction and may synergize with autonomic and sleep-related stressors (Mao et al., 2024). Similarly, arteriosclerosis, linked to increased arterial stiffness in OSA, amplifies vascular damage (Lee et al., 2021). These cerebrovascular findings are consistent with the broader systemic vascular consequences of OSA, in which intermittent hypoxia, inflammation, and endothelial dysfunction contribute to atherosclerotic and coronary vascular injury (Tetaj et al., 2026). Higher statin use in the CSVD group, likely reflecting clinical management of cardiovascular risk, may further modulate lipid profiles and inflammation (Diamantis et al., 2017; Soulaidopoulos et al., 2018). This finding underscores the importance of early screening and intervention in OSA patients to mitigate CSVD risk.

## 5 Limitation

This study has several limitations. First, as a retrospective cross-sectional analysis, causal inference cannot be drawn, and the temporal relationship between nocturnal minimum heart rate and CSVD cannot be determined. Second, despite adjustment for multiple confounders, residual confounding from unmeasured clinical factors cannot be fully excluded. Third, single-center recruitment may restrict the generalizability of our observations. Fourth, data on CPAP-related treatment were not available in this cohort. Future prospective studies are required to validate our findings.

## 6 Conclusion

This study demonstrated that an elevated nocturnal minimum heart rate, a readily obtainable polysomnographic marker, is independently associated with CSVD in patients with OSA. These findings underscore the potential value of incorporating autonomic indicators into risk stratification for OSA and highlight the need for longitudinal research to determine whether nocturnal heart rate predicts CSVD progression and whether OSA treatment modifies this relationship.

## Data Availability

The datasets presented in this article are not readily available because “The datasets analyzed for this study can be found in the article and its Supplementary material. Additional data are available from the corresponding author upon reasonable request”. Requests to access the datasets should be directed to Mingming Zhao, topabigale@163.com.
