# Supplementary material for: Association between minimum nocturnal heart rate and cerebral small vessel disease in patients with obstructive sleep apnea
Source: Front Aging Neurosci. 2026 Sep 15;18:1848124. doi: 10.3389/fnagi.2026.1848124 (PMC13619923; doi:10.3389/fnagi.2026.1848124)
Supplement: Supplementary file 1 [file Supplementary_file_1.docx]

Supplementary Material

# 1 Supplementary Figures and Tables

## 1.1 Supplementary Tables

Supplementary TableS1. Distribution of CSVD Imaging Markers among 265 Patients

| CSVD Imaging Marker | n (%) |
| --- | --- |
| White matter hyperintensities | 250 (94.3) |
| Lacunes of presumed vascular origin | 15 (5.7) |
| Brain atrophy | 14 (5.3) |
| Enlarged perivascular spaces | 5 (1.9) |
| Cerebral microbleeds | 3 (1.1) |
| Recent small subcortical infarcts | 2 (0.8) |

CSVD: cerebral small vessel disease.

Supplementary Table S2. WMH severity graded by the Fazekas scale in CSVD patients (n = 265).

| Fazekas grade | n | % |
| --- | --- | --- |
| 0 | 12 | 4.5 |
| 1 | 239 | 90.2 |
| 2 | 13 | 4.9 |
| 3 | 1 | 0.4 |

WMH, white matter hyperintensity; CSVD, cerebral small vessel disease.

Supplementary TableS3. Variance Inflation Factors (VIF) for Model Predictors

| Variables | VIF |
| --- | --- |
| LDL-C | 18.412 |
| ApoB | 15.322 |
| TC | 9.647 |
| ApoA1/B | 6.578 |
| ALT | 7.001 |
| AST | 6.722 |
| sleep onset latency | 32.200 |
| stage N1 latency | 30.347 |
| wake time | 5.992 |
| wake time after sleep onset | 5.516 |
| HDL-C | 4.025 |
| ApoA1 | 4.207 |
| ALB | 2.459 |
| TP | 2.425 |
| Age | 2.098 |
| percentage of stage N1 in TST | 1.855 |
| Sex | 1.754 |
| ChE | 1.625 |
| number of awakenings | 1.539 |
| UA | 1.413 |
| stage N3 latency | 1.466 |
| percentage of stage N3 in TST | 1.323 |
| Arteriosclerosis/Arterial plaque | 1.358 |
| Beta-blockers | 1.272 |
| minimum heart rate | 1.271 |
| Marry | 1.211 |
| PLM index in stage REM | 1.212 |
| hypertension | 1.203 |
| Urea | 1.202 |
| Statin Use | 1.194 |
| Diabetes | 1.159 |
| Smoke | 1.131 |
| number of arousals caused by limb movement during REM sleep | 1.097 |
| TBA | 1.086 |

TC: total cholesterol; TG: triglyceride; LDL-C: low-density lipoprotein cholesterol; HDL-C: high-density lipoproteincholesterol; ApoA1: apolipoprotein A1; ApoB: apolipoprotein B; CREA: creatinine; UA: uric acid; ALT: alanine aminotransferase; AST: aspartate aminotransferase; TP: total protein; ALB: albumin; ChE: cholinesterase; TBA: total bile acids. TST: total sleep time; REM: rapid eye movement, PLM: periodic leg motion.

Supplementary Table S4. Sensitivity analysis excluding statin users (CSVD ~ age + minimum nocturnal heart rate).

| Model | Variable | OR | 95% CI | *P* | n |
| --- | --- | --- | --- | --- | --- |
| All patients | Age | 1.093 | 1.074–1.114 | <0.001 | 630 |
| All patients | Minimum heart rate | 1.024 | 1.007–1.041 | 0.006 | 630 |
| Statin users excluded | Age | 1.086 | 1.065–1.108 | <0.001 | 543 |
| Statin users excluded | Minimum heart rate | 1.021 | 1.003–1.039 | 0.019 | 543 |

OR, odds ratio; CI, confidence interval; CSVD, cerebral small vessel disease.

Supplementary Table S5. Minimum nocturnal heart rate and CSVD proportion stratified by OSA severity.

| OSA severity (AHI) | n | CSVD, n (%) | Min HR, all, median [IQR] | Min HR, non-CSVD, median | Min HR, CSVD, median |
| --- | --- | --- | --- | --- | --- |
| Mild (5–15) | 59 | 23 (39.0) | 55.0 [50.0, 61.0] | 55.5 | 54.0 |
| Moderate (15–30) | 107 | 41 (38.3) | 58.0 [50.5, 64.0] | 57.5 | 59.0 |
| Severe (≥30) | 464 | 201 (43.3) | 59.0 [53.0, 65.0] | 58.0 | 61.0 |

AHI, apnea–hypopnea index (events/h); HR, heart rate (bpm); CSVD, cerebral small vessel disease; IQR, interquartile range.

Supplementary Table S6. Sensitivity analysis: multivariable model with and without adjustment for overall AHI (n = 630).

| Model | Variable | OR | 95% CI | *P* |
| --- | --- | --- | --- | --- |
| A. Primary model | Minimum heart rate | 1.023 | 1.006-1.040 | 0.008 |
|  | Age | 1.095 | 1.071-1.118 | <0.001 |
|  | Sex | 1.324 | 0.784-2.233 | 0.294 |
|  | Marital status | 0.935 | 0.541-1.615 | 0.809 |
|  | Smoke | 1.071 | 0.673-1.704 | 0.774 |
|  | Statin use | 1.198 | 0.706-2.034 | 0.504 |
|  | Beta-blocker use | 0.922 | 0.407-2.088 | 0.846 |
|  | Diabetes | 1.038 | 0.524-2.058 | 0.915 |
|  | Hypertension | 1.119 | 0.740-1.692 | 0.593 |
| B. Primary model + AHI | Minimum heart rate | 1.022 | 1.004–1.039 | 0.014 |
|  | Age | 1.097 | 1.073–1.121 | <0.001 |
|  | Sex | 1.289 | 0.758–2.192 | 0.348 |
|  | Marital status | 0.926 | 0.535–1.602 | 0.783 |
|  | Smoke | 1.059 | 0.665–1.687 | 0.809 |
|  | Statin use | 1.190 | 0.700–2.021 | 0.520 |
|  | Beta-blocker use | 0.909 | 0.401–2.057 | 0.818 |
|  | Diabetes | 1.065 | 0.538–2.107 | 0.857 |
|  | Hypertension | 1.107 | 0.732–1.675 | 0.630 |
|  | AHI | 1.003 | 0.995–1.010 | 0.482 |

OR, odds ratio; CI, confidence interval; AHI, apnea–hypopnea index; CSVD, cerebral small vessel disease.
